# Role of Magnesium and the Effect of Surface Roughness on the Hydroxyapatite-Forming Ability of Zirconia Induced by Biomimetic Aqueous Solution Treatment

**DOI:** 10.3390/ma13143045

**Published:** 2020-07-08

**Authors:** Hasnat Zamin, Takeshi Yabutsuka, Shigeomi Takai, Hiroshi Sakaguchi

**Affiliations:** 1 Graduate School of Energy Science, Kyoto University, Yoshida-Honmachi, Sakyo-ku, Kyoto 606-8501, Japan; zaminhasnat@gmail.com (H.Z.); stakai@energy.kyoto-u.ac.jp (S.T.); 2 Institute of Advanced Energy, Kyoto University, Gokasho, Uji, Kyoto 611-0011, Japan; sakaguchi.hiroshi.7z@kyoto-u.ac.jp

**Keywords:** tetragonal zirconia polycrystal, etching, calcium phosphate, magnesium, hydroxyapatite, in vitro bioactivity, adhesive strength

## Abstract

Zirconia is a well-known bioceramic for dental and orthopedic applications due to its mechanical and aesthetic properties. However, it lacks sufficient bioactivity to bond with the living bone. This study was aimed to induce bioactivity to tetragonal zirconia polycrystal (3Y-TZP) by simple biomimetic aqueous solution treatment. First, hydrofluoric acid (HF) etching was performed to enhance the surface roughness of the 3Y-TZP surface. Then, the samples were treated with two types of aqueous solutions containing calcium and phosphate ions (Ca-P solutions); one solution additionally contained magnesium (Mg) ions and the other without Mg ions. Finally, hydroxyapatite (HAp)-forming ability was evaluated by the conventional simulated body fluid (SBF) test, and the effect of Mg ions on the adhesive strength of the HAp layer to the roughened 3Y-TZP surface was also investigated. The results concluded that there were no noticeable differences in the effect of Mg ions on the HAp-forming ability, and both types of solution treatments resulted in dense HAp formation in 1 day SBF immersion. However, incorporation of Mg ions in one of the Ca-P solutions significantly improved the adhesive strength of the HAp layer to the HF-etched 3Y-TZP substrate compared to the Ca-P solution with no Mg ions.

## 1. Introduction

Zirconia is a highly considered biocompatible ceramic, also known as the “ceramic steel” due to its outstanding mechanical properties [1,2]. This makes it highly suitable for orthopedic applications such as femoral heads and acetabular cups, and, in dentistry, it is used as crowns, implants, and abutments [2,3]. Zirconia exists in three crystal forms monoclinic, cubic, and tetragonal. Pure zirconia is in the monoclinic phase at room temperature. When mixed with 2–3 mol% yttria, zirconia is stabilized into the tetragonal phase and known as yttria-stabilized zirconia or tetragonal zirconia polycrystal (3Y-TZP). 3Y-TZP shows excellent fracture toughness and flexural strength, which is due to the phase transformation toughening that enhances resistance to the propagating crack. As the 3Y-TZP surface is subjected to stress, the crack stress field leads to phase transformation from tetragonal to monoclinic. This phase change causes a volumetric change that seals the advancing crack [1,4]. Another noteworthy characteristic of zirconia is its aesthetic value of being white in color, which is in contrast to metals that can cause metallic discoloration [5,6].

A bioactive material is defined as “a material that elicits a specific biological response at the interface of the material, which results in the formation of a bond between the tissues and the material” [7]. When a bioinert material is grafted into the bone defect, it spontaneously gets coated with non-calcified fibrous tissues. This isolates it from the surrounding living tissue, which is a normal immune response of the human body against exogenous substances [8,9]. Hench discovered 45S5 Bioglass in the year, 1969, which had the ability to form an interfacial bond between the tissue and the implant. This interesting feature of bioactive glasses as described by Hench is due to the formation of carbonated hydroxyapatite when exposed to the physiological fluid [10]. Since then, a great deal of effort has been put to the development of novel bioactive materials. In the field of orthopedics, ceramic biomaterials such as bioglass and calcium phosphate-based materials such as hydroxyapatite (HAp) are highly considered due to their inherent bioactive nature [11,12]. Hence, one of the best approaches to induce bioactivity to a bioinert material is to coat it with suitable bioactive material, such as HAp.

For bioinert ceramics and metallic materials, various techniques such as sputtering, electrophoretic deposition, and plasma spraying have been employed to provide a suitable HAp coating. However, these methods require a high-temperature treatment or are expensive [13,14]. High-temperature treatment starting from 850 °C results in dehydroxylation of HAp that creates vacancies in the lattice and leads to the formation of oxyhydroxyapatite. After further heating beyond the temperature of 1050 °C, HAp starts to decompose into other variants of calcium phosphates such as tetracalciumphosphate, tricalciumphosphate, and CaO [15]. These variants have faster dissolution and degradation rates compared to HAp which decreases the chemical stability in the body for a longer period [16].

Kukobo et al. developed simulated body fluid (SBF), which has an ionic concentration and a pH similar to human blood plasma. SBF can mimic the formation of HAp on the surface of a bioactive material similar to the living body. Kukobo’s method has widely been used to test and predict the bioactivity of a material [17,18]. Biomimetic calcium phosphate-based coatings utilizing SBF or modified SBF treatments are the simplest methods to impart bioactivity to bioinert material as it requires low processing temperatures and the HAp formed in biomimetic coating resembles the bone’s HAp [19,20,21,22,23].

Various studies have been performed on in-vitro bioactivity assessment of surface coated zirconia or zirconia composites in SBF. These studies reported bone-like apatite formation ranging from 3 days to 4 weeks of immersion in the SBF [24,25,26,27,28,29,30]. Magnesium (Mg) is a biofunctional cation present, abundantly, in the human body, which promotes bone cells activation and proliferation. It also influences bone strength, mineralization, and growth [31,32]. Many studies have also investigated the role of Mg in enhancing osteointegration [33,34,35,36].

Herein, the authors report the fabrication of the bioactive 3Y-TZP by treating it with calcium and phosphate ions-containing biomimetic aqueous solution (Ca-P solution), which also included Mg ions in a very short immersion period of 1 day in the SBF. Hydrofluoric acid (HF) etching was performed on the 3Y-TZP surface to enhance the surface roughness. Then the samples were treated with two different types of Ca-P solutions one with and the other without Mg ions, to analyze the effect of Mg ions on the HAp layer formed in the SBF. SBF immersion test was carried out in order to investigate the HAp-forming ability of thus-treated 3Y-TZP. Furthermore, the effect of the Mg ion on the adhesive strength of the HAp layer on the etched 3Y-TZP surface was also investigated.

## 2. Materials and Methods

### 2.1. Outline of the Experimental Procedure

The experimental procedure consisted of the following steps. First, HF etching was performed on the polished 3Y-TZP surface. Then, 3Y-TZP samples were subjected to two types of Ca-P solution treatments. SBF immersion test of both types of Ca-P solution treated samples was carried to check the HAp-forming ability. Finally, the adhesive strength of the formed apatite layer was evaluated.

#### 2.1.1. Fabrication of the Roughened 3Y-TZP Samples

Dental-grade 3Y-TZP (KZR-CAD ZR, Yamakin Co. Ltd., Osaka, Japan) was cut into 2 mm thick samples using a diamond wheel. Sintering was performed according to prerequisite instructions provided by the manufacturer, the 3Y-TZP samples were sintered at peak sintering temperature of 1450 °C, the sintering cycle consisted of four steps. First, the samples were heated from room temperature to 500 °C in 2 h (≈4 °C·min^−1^). Next, the temperature was raised from 500 °C to the peak temperature in 4.5 h (≈3.5 °C·min^−1^). Then, dwelling at the peak sintering temperature was done for two hours. Finally, the samples were gradually allowed to cool down. After sintering the samples were polished using #400 abrasive-coated paper. Then, HF etching was performed by immersing the 3Y-TZP samples in HF (~55% concentration, Stella Chemifa Co., Ltd. Izumi factory, Osaka, Japan) at room temperature for 10 min. The obtained samples were ultrasonically cleansed for 10 min each in acetone and distilled water, respectively, and were air-dried. Finally, surface analysis and surface roughness measurement of the samples before and after the HF etching treatment as described in the Section 2.2.1.

#### 2.1.2. Ca-P Solution Treatments

Two types of Ca-P solutions were prepared in distilled water containing K_2_HPO_4_·3H_2_O, MgCl_2_·6H_2_O and CaCl_2_ as mentioned in Table 1 at 25.0 °C and a pH 8.2 using tris(hydroxymethyl)aminomethane (THAM; Hayashi Pure Chemical Ind., Ltd., Osaka, Japan). One solution containing Mg ions was named as “Mg1.5” solution while the other which did not contain Mg ions was named as “Mg0” solution. The concentration of the “Mg1.5” solution contained similar concentrations of Ca^2+^, Mg^2+^, and phosphate ions as compared to the conventional SBF, while “Mg0” solution only contained Ca^2+^ and phosphate ions also similar to conventional SBF [17,18]. The rest of the ions were removed from the solution to distinguish the effect of Mg ions on the HAp formed in the SBF. The HF-etched 3Y-TZP samples were soaked in these solutions at 70 °C for 1 day. After 1 day, white particles were observed to be precipitated in both types of solutions, which were denoted as “Mg0” and “Mg1.5” particles, respectively. Then, the 3Y-TZP samples were removed from the solution and the remaining white precipitates were collected by vacuum filtration using 0.025 μm membrane filter (Merck Millipore, Burlington, MA, USA).

#### 2.1.3. HAp-Forming Ability

After the Ca-P solution treatments the 3Y-TZP samples were immersed in the SBF to test the HAp-forming ability for 1 and 7 days. The SBF solution was replenished after 3 days. The SBF was prepared by the method reported by Kokubo et al. [17,18] and buffered at pH = 7.4, 36.5 °C by disillusion of THAM.

#### 2.1.4. Analysis

The surface of the samples mentioned in the Section 2.1.1 and Section 2.1.3 and collected Ca-P particles mentioned in Section 2.1.2 were analyzed by high-vacuum field emission scanning electron microscopy (SEM; SU6600, Hitachi High-Technologies Corporation, Tokyo, Japan), energy dispersive X-ray spectrometry (EDS; XFlash^®^ 5010, Bruker, Billerica, MA, USA), thin film X-ray diffraction (XRD; Rint 2500, Rigaku Corporation, Tokyo, Japan), and Fourier transform infrared spectroscopy (FTIR; FT-720, Horiba, Ltd., Kyoto, Japan). Before SEM and EDX observation the samples were coated with Au using sputtering method. For SEM analysis acceleration voltage of 20 kV, emission current of 32 μA and secondary electron detector was used. For the EDX an acceleration voltage of 20 kV and emission current of 32 μA was used. After the SEM and EDX analysis, XRD measurement of the same samples were performed to compare the surface morphology and composition with corresponding crystalline phase from 2θ range of 20°–80° using angle step of 1°·min^−1^. FTIR analysis was done using Attenuated total reflectance (ATR).

### 2.2. Evaluation of Materials Properties

#### 2.2.1. Surface Roughness Measurement

Surface roughness of the surface of the samples before and after the HF etching mentioned in Section 2.1.1 was compared by ultra-precision point autofocus laser probe 3D measuring instrument (NH-3SP, Mitaka Kohki Co., Ltd., Tokyo, Japan). In this measurement, heights at 10,021 points in 10 by 10 μm^2^ of base area were probed and root mean square surface roughness (S_q_) was calculated from the probed information. One sample was used for each condition.

#### 2.2.2. Evaluation of Ca/P Atomic Ratio and Mg Release

Measurement of inductively coupled plasma atomic emission spectroscopy (ICP; ICP7510, Shimadzu Corporation, Kyoto, Japan) was carried out to find out the concentration (ppm·mL^−1^) of the Ca, P, and Mg present in the Ca-P particles precipitated in the “Mg0” or “Mg1.5” solution. The obtained results were used to calculate the Ca/P and Mg/Ca ratio for each type of mentioned particles and compared with Ca/P and Mg/Ca ratio of the commercially obtained HAp (Fujifilm Wako Pure Chemical, Osaka, Japan). For this, both types of Ca-P particles along with commercially obtained HAp were dissolved 1 M HCl (Fujifilm Wako Pure Chemical, Osaka, Japan) at a concentration of 0.1 mg·mL^−1^. Three samples of solutions for each type of the mentioned particles were used for concentration measurement of the respective ions. Release of Mg ions from “Mg1.5” particles in 0.01 M phosphorus buffered saline (PBS, pH = 7.2~7.4 at 25 °C, Fujifilm Wako Pure Chemical) was also measured. Similarly, the “Mg1.5” particles were dispersed in PBS at a concentration of 0.1 mg·mL^−1^ and stored at the physiological temperature of 36.5 °C. The particles were filtered using vacuum filtration using 0.025 μm membrane filter and concentration of Mg ions released in PBS was measured at the various time period. Three samples were prepared for measurement of concentration of Mg ions at each time period. One-way analysis of variance (ANOVA) followed by Tukey’s multiple comparison tests was carried out to calculate *p* values and to evaluate significant difference between Ca/P ratios for HAp, “Mg0”, and “Mg1.5” particles.

#### 2.2.3. Adhesive Strength Test

The bonding strength between the HF-etched 3Y-TZP samples and the formed HAp film was examined and compared with polished 3Y-TZP samples after 14 days SBF immersion by a modified ASTM C633 method [37,38,39]. Two sets each containing three samples for polished 3Y-TZP samples treated with “Mg0” and “Mg1.5” solutions, respectively, and two sets each containing five samples for HF-etched 3Y-TZP samples treated with “Mg0” and “Mg1.5” solutions, respectively, were tested. The surfaces of the samples were attached to the SUS jigs (10 by 10 mm^2^) using Araldite^®^ glue (Nichiban Co., Ltd., Tokyo, Japan) and the tensile load was applied at 1 mm·min^−1^ of cross-head speed until fracture occurred at the HAp film and the samples interface using a universal testing machine (Model AGS-H Autograph, Shimadzu Corporation, Kyoto, Japan). Finally, the fractured surface was analyzed by SEM and EDX surface scanning to find out the mechanism of the fracture. One-way ANOVA followed by Tukey’s multiple comparison tests was carried out to calculate *p* values and to evaluate significant difference between adhesive strength for each type of the above-mentioned conditions.

## 3. Results and Discussion

**Figure 1:**
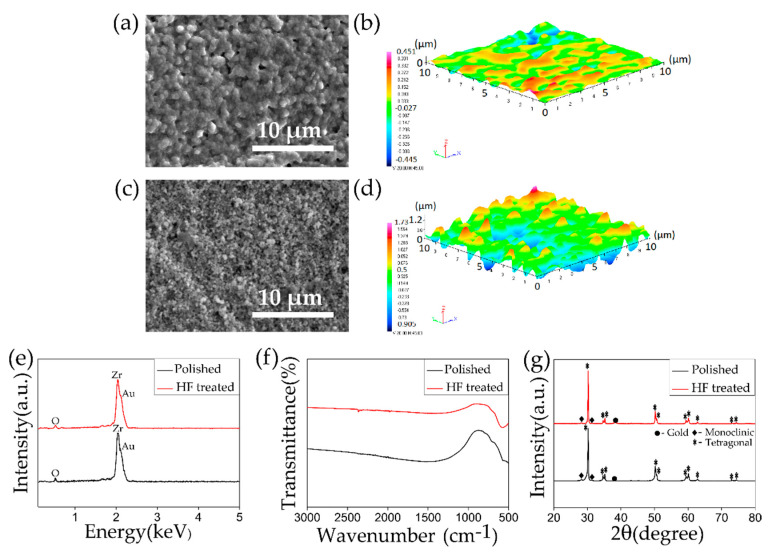
(**a**,**c**) SEM images and (**b**,**d**) 3D images of (**a**,**b**) polished and (**c**,**d**) hydrofluoric acid (HF)-treated tetragonal zirconia polycrystal (3Y-TZP) surface. (**e**) EDX, (**f**) FTIR, and (**g**) XRD plots comparing 3Y-TZP samples before (polished) and after HF etching.

Figure 1 compares the effect of HF etching on the 3Y-TZP surface. In this study, the samples were subjected to HF etching treatment for 10 min aimed to enhance the roughness of the 3Y-TZP surface. As it can be observed from the SEM and 3D images in Figure 1a–d that the surface got highly roughened after the treatment. The S_q_ value rose from ~0.126 μm to ~0.401 μm before and after the HF etching. In the EDX spectra as shown in Figure 1e peaks representing Zr and O was detected and in the FTIR spectra, as shown in Figure 1f, peak at ~500 cm^−1^ was observed, which is fundamental infrared frequency attributable to the XRD patterns as depicted in Figure 1g, both before and after the HF etching revealed the presence of the tetragonal phase predominantly, a small characteristic peak representing the monoclinic phase was detected at 28.1°. 3Y-TZP is highly chemically stable, the peaks in the EDX, FTIR, and XRD plots before and after the HF treatment showed no significant change, which means that there was no change in the elemental composition. Noro et al. showed the effect of various surface treatments on the roughness of the 3Y-TZP surface. It was observed that a combination of sandblasting and HF etching (~44%) for 15 min resulted in a highly roughened surface with superhydrophilic properties [40]. Noro’s work and the experimental methodology in the present study shows the effectiveness of the HF treatment in enhancing surface roughness of the 3Y-TZP.

**Figure 2:**
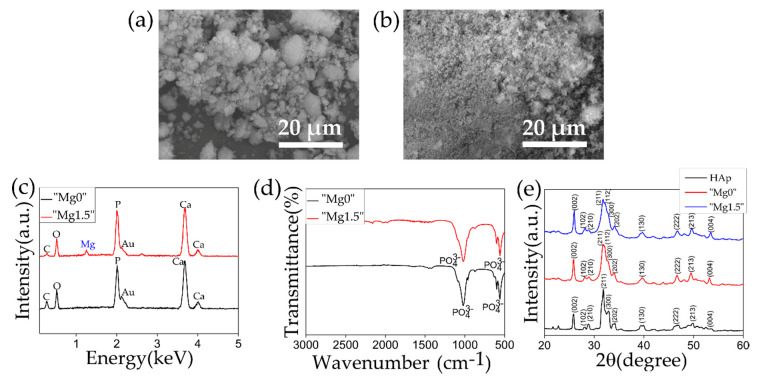
SEM images of (**a**) “Mg0” and (**b**) “Mg1.5” particles. (**c**) EDX and (**d**) FTIR plots comparing properties of “Mg0” and “Mg1.5” particles. (**e**) XRD plots comparing properties of “Mg0” and “Mg1.5” particles with commercially obtained HAp.

Figure 2 represents the SEM images, EDX, FTIR, and XRD plots of both types of Ca-P particles. In the SEM images, coarse particles with uneven shape and size were observed for “Mg0” particles while fine spherical particles having size around 1–2 μm were observed in the case of “Mg1.5” particles. As was observed from strong peaks from PO_4_^3−^ of P=O stretching at 1050 cm^−1^ and 580 cm^−1^ in FTIR spectra and strong Ca and P peaks in the EDX spectra, both types of particles were composed of calcium phosphate. A Mg peak was observed in the EDX spectrum only for the case of “Mg1.5” particles that indicated the incorporation of Mg in the particles. In the Figure 2e the XRD patterns of “Mg0” and “Mg1.5” were compared with the commercially obtained HAp. Both “Mg0” and “Mg1.5” particles patterns resembled that of typical HAp, however, slightly broader peaks suggested that these particles were slightly less crystalline compared to HAp.

The formation of HAp in an aqueous solution from the constituent ions can be described by the following chemical equilibrium.

10Ca^2+^ + 6PO_4_^3-^ + 2OH^−^ ⇌ Ca_10_(PO_4_)_6_(OH)_2_ (1)

Considering the equation ionic activity product (IP) of the apatite in the solution can be described by the following formula, where “γ” is the activity coefficient and “[ ]” is the concentration of each ion.

IP = (γCa^2+^)^10^(γPO_4_^3−^)^6^(γOH^−^)^2^ × [Ca^2+^]^10^[PO_4_^3−^]^6^[OH^−^]^2^ (2)

Conventional SBF at a physiological condition, i.e., pH 7.40, and 36.5 °C, is supersaturated with respect to HAp. However, because of the high energy obstacles with respect to the HAp formation, the HAp formation is only induced in certain active surfaces as in the case of bioactive materials [41]. The rate of precipitation and the properties of precipitated Ca-P particles such as crystallinity and phase are strongly dependent on the physical parameters such as temperature and pH, and, also, the concentration of the constituent ions in the aqueous solution. In this work, the concentration of the respective ions (Ca, Mg, and phosphate) in the both types of aqueous solution were kept the same as the conventional SBF. The pH of the aqueous solution was raised to 8.2, which presumably resulted in the increase of IP value because of the increase in the OH^−^ concentration. Initially, the temperature was maintained at 25 °C during the preparation of the solution to slow the precipitation of particles and then raised and held at 70 °C to accelerate the precipitation. This method successfully resulted in the formation of Ca-P particles in a 1 day time period from both types of solutions.

HAp crystallization is greatly affected by the presence of additional ion besides Ca^2+^ and PO_4_^3−^ or HPO_4_^2−^ such as Mg which results in the reduction of Ca/P ratio and decrease in crystal size. Bigi et al. reported that the decrease in the crystal size was highly significant even at the lower Mg percentage, and crystal size decreases with the increase in Mg concentration up to 35 atomic percentage in respect of the total metal ions. At a concentration between 35 to 50 atomic percentage of Mg, the particles are amorphous and more than 50 percent results in the formation of different crystalline phases [42].

**Figure 3:**
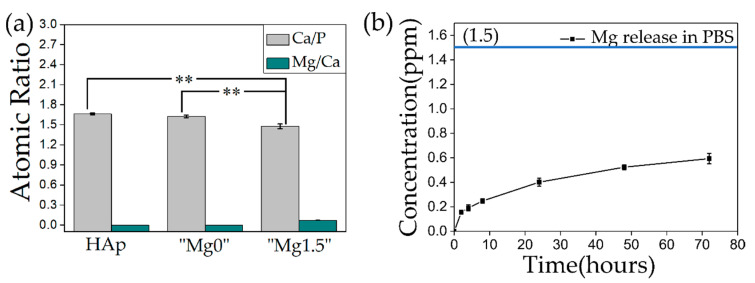
(**a**) Comparison of Ca/P and Mg/Ca atomic ratio of “Mg0” and “Mg1.5” particles with commercially available HAp particles. In (**a**), the symbol “**” indicates *p* < 0.01, and no symbol indicates *p* > 0.05 by one-way ANOVA followed by the Tukey’s tests. (**b**) release behavior of Mg ions from “Mg1.5” particles in the PBS. In (**b**), blue straight line shows 1.503 ppm which was assumed concentration if all the Mg contained in the “Mg1.5” particles had been released to the phosphorus buffered saline (PBS).

Figure 3 shows the results of ICP measurements. ICP measurement was performed to measure the concentration of Ca/P and Mg/Ca ratios in both types of Ca-P particles and compared against the commercially available HAp as represented in Figure 3a. The error bar shows the standard deviation obtained from the three samples used for concentration measurement for each type of particles. By applying one-way ANOVA followed by the Tukey’s tests, Ca/P ratios of HAp and “Mg0” particles showed no statistical significance (*p* > 0.05). In contrast, statistical comparison of Ca/P ratio of HAp or “Mg0” particles with “Mg1.5” particles showed statistical significance (*p* < 0.01). It was difficult to compare the statistical significance of Mg/Ca ratio due to significantly low value and detection limitation. Mg ions incorporation in “Mg1.5” particles resulted in the significant decrease of Ca/P ratio. Compared against HAp Ca/P atomic ratio of 1.66, for “Mg0” and “Mg1.5” particles Ca/P ratio was found to be ~1.625 and ~1.478, respectively. For “Mg1.5” particles, the Mg/Ca atomic ratio was found to be ~0.0728 which is low, however, it significantly decreased the particle size as observed from the SEM images shown in Figure 2a,b. As it was mentioned in the explanation of the XRD peaks of both “Mg0” and Mg”1.5” particles in Figure 2e, the slight decrease in the crystallinity of the particles was attributed to the decreased Ca/P ratio as compared to commercial HAp as represented in Figure 3a.

Similarly, the release of Mg ions was measured from 0.1 mg of “Mg1.5” particles in 1 mL of PBS (at 36.5 °C) starting from 2 h to 3 days and at different time periods in between, as shown in Figure 3b. The error bar represents the standard deviation obtained from three samples prepared for measurement of the concentration of Mg ions released from “Mg1.5” particles in PBS at respective time periods. When the same amount (0.1 mg) of “Mg1.5” particles were dissolved in same volume (0.1 mL) of 1M HCl to evaluate total Mg contained in the “Mg1.5” particles, the concentration of Mg ions in “Mg1.5” particles was found to 1.503 ppm. As it can be observed from the graph that a significant amount of Mg ions was released in 2 h and about ~26.6% of Mg ions were released from the particles in the PBS in 1 day and then Mg ions were continuously released. This result indicated that the “Mg1.5” particles showed sustained release of Mg ions in biological environment.

**Figure 4:**
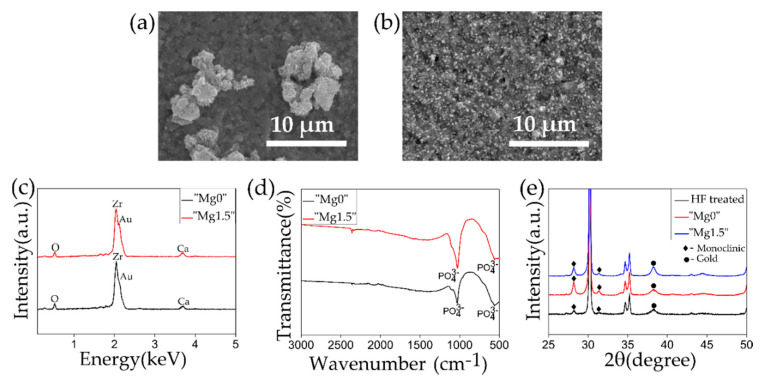
SEM images of HF-etched 3Y-TZP samples after (**a**) “Mg0” and (**b**) “Mg1.5” solution treatments. (**c**) EDX and (**d**) FTIR comparing properties of the HF-etched 3Y-TZP samples after “Mg0” or “Mg1.5” solution treatments. (**e**) XRD plots of HF-etched 3Y-TZP samples before and after “Mg0” or “Mg1.5” solution treatments.

Figure 4 shows the SEM images, EDX and FTIR plots on the surface of the HF-etched 3Y-TZP samples after the 1 day Ca-P solution treatments. As it can be observed in the SEM images, “Mg0” solution treatment resulted in the deposition of coarse particles, of uneven shape and size in clusters, while in the case of “Mg1.5” solution treated samples, the 1–2 µm sized particles were homogenously deposited throughout the surface of the substrate. Such difference in the particle size was corresponded to the results of comparative observation of the filtered “Mg0” and “Mg1.5” particles shown in Figure 2. Small Ca peak was detected from both types of particles in the EDX spectra while P peak was difficult to distinguish as it coincided with the Zr peak. In the FTIR spectra for both types of solution treated samples, the strong peak of P=O stretching from PO_4_^3−^ at 1050 cm^−1^ and 580 cm^−1^ was observed. Comparing the XRD plots before (after HF treatment) and after the Ca-P solutions treatments, no significant difference was observed. The characteristics peaks of HAp at 26° and 32° were not observed, which was due to the formation of isolated and fewer number of Ca-P particles rather than a continuous layer leading to insufficient surface coverage. On the other hand, the peak at 28.1° representing the monoclinic phase of zirconia became more prominent. It is considered that this was due to the exposure to water, which caused tetragonal to monoclinic transformation in zirconia, a phenomenon known as low-temperature degradation [43,44]. The Au peak detected in the XRD is from the deposition of Au layer for the SEM observation of the same samples. In this study, both types of Ca-P solution treatments were designed to efficiently deposit Ca-P particles on the substrate. It is noteworthy to observe that the inclusion of Mg led to the nucleation of small and fine particles homogeneously throughout the substrate.

**Figure 5:**
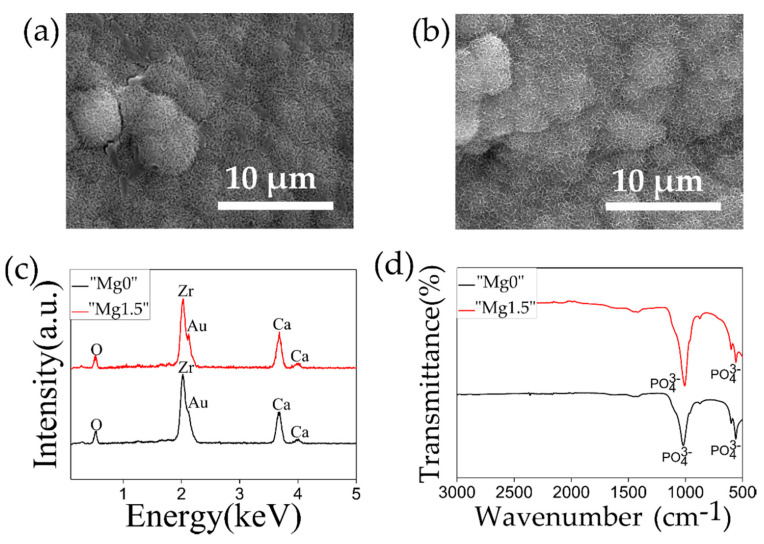
SEM images of (**a**) “Mg0” solution and (**b**) “Mg1.5” solution-treated 3Y-TZP samples after 1 day SBF immersion. (**c**) EDX and (**d**) FTIR plots comparing properties of “Mg0” solution and “Mg1.5” solution-treated 3Y-TZP samples after 1 day SBF immersion.

**Figure 6:**
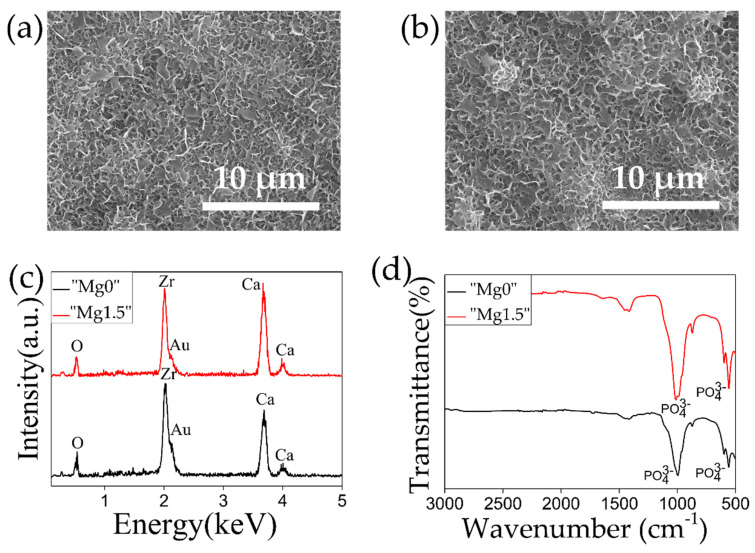
SEM images of (**a**) “Mg0” solution and (**b**) “Mg1.5” solution-treated 3Y-TZP samples after 7 days SBF immersion. (**c**) EDX and (**d**) FTIR plots comparing properties of “Mg0” solution and “Mg1.5” solution-treated 3Y-TZP samples after 7 days SBF immersion.

Figure 5 and Figure 6 shows SEM images, EDX, and FTIR plots of both the types of HF-etched and subsequently Ca-P solution-treated 3Y-TZP samples after 1 day and 7 days of SBF immersion. From the SEM images, it can be observed that flake-like crystallites representing bone-like apatite, covered the whole surface of the 3Y-TZP samples for both types of Ca-P solution treatments irrespective of the Mg inclusion in the solution. Similarly, P=O stretching peak at 1050 cm^−1^ and 580 cm^−1^ in the FTIR plots and high intensity Ca peaks observed in the EDX plots were observed for both types of solution treated samples.

**Figure 7:**
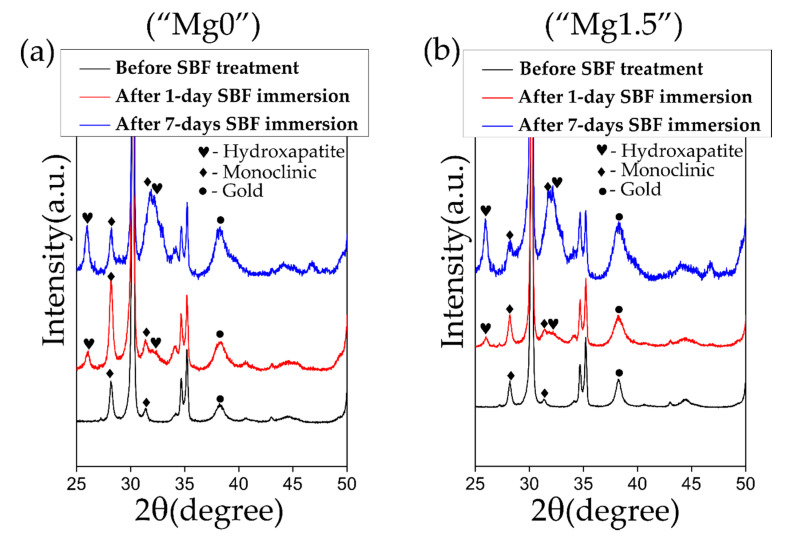
XRD plots comparing properties of (**a**) “Mg0” solution and (**b**) “Mg1.5” solution-treated 3Y-TZP samples before and after 1 day or 7 days SBF immersion.

Figure 7 compares XRD plots of 3Y-TZP samples after 1 day and 7 days SBF immersion with both types of “Mg0” solution and Mg”1.5” solutions-treated samples to clarify the formation of HAp. In the XRD plots, characteristics peaks of HAp at 26° and 32° were detected for both types of solution treated samples for 1 day SBF immersion which got further intensified after 7 days SBF immersion. This means that both the types of solution treatments induced high HAp-forming ability to 3Y-TZP in 1 day SBF immersion. Compared to a similar work [30], in the present study short induction time for the HAp formation can be attributed to good deposition of Ca-P particles. These particles increased the degree of supersaturation of the Ca ions in the SBF solution at the vicinity of the 3Y-TZP surface accelerating the formation of the HAp.

**Figure 8:**
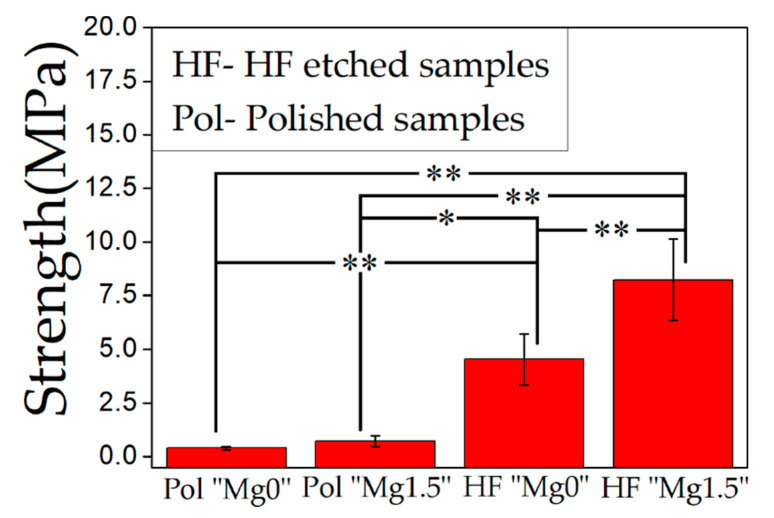
Comparison of adhesive strength of the HAp layer on only polished (Pol) and HF-etched and subsequently “Mg0” solution-treated and “Mg1.5” solution-treated 3Y-TZP formed by 14 days SBF immersion. The symbol “**” indicates *p* < 0.01, “*” indicates *p* < 0.05 and the no symbol indicates *p* > 0.05 by one-way ANOVA followed by the Tukey’s tests.

Figure 8 shows the average adhesive strength of the HAp layer to the HF-etched 3Y-TZP samples compared to fine polished 3Y-TZP samples. In this figure “Pol” refers to polished and “HF” refers to HF etched 3Y-TZP samples. The error bar in the figure represents the standard deviation. By applying one-way ANOVA followed by the Tukey’s tests, statistical comparison of adhesive strength of Pol “Mg0” and Pol “Mg1.5” (*p* > 0.05) samples showed no statistical significance. Whereas, adhesive strength of HF “Mg1.5” samples compared statistically with Pol “Mg0”, Pol “Mg1.5”, or HF “Mg0” (*p* < 0.01) showed statistical significance. Statistical comparison of adhesive strength of Pol “Mg0” and HF “Mg0” (*p* < 0.01) or Pol “Mg1.5” and HF “Mg0” (*p* < 0.05) also showed statistical significance. In the case of the polished 3Y-TZP samples, the adhesive strength was found to be very low irrespective of the condition of Ca-P solution treatments. While the HF treatment resulted in a significant increase in the adhesive strength of the HAp layer to the 3Y-TZP surface compared to polished samples, for both types of Ca-P solution-treated samples which was due to improvement of interlocking effect [45]. The important point to note is that the adhesive strength of the HAp layer to the HF-etched 3Y-TZP surface for “Mg1.5” solution treated samples were also significantly higher compared to “Mg0” solution treated HF-etched 3Y-TZP samples. This concludes that HF treatment contributed to the improvement of the adhesive strength of the HAp film. Incorporation of Mg ions in the Ca-P solution further contributed to improvement of the adhesive strength of the HAp layer. However, this contribution of Mg ion was very small or unnoticeable without the HF treatment. To understand the difference in the mechanism of failure, SEM and EDX analysis of the fractured surface of HF-etched 3Y-TZP samples were performed.

**Figure 9:**
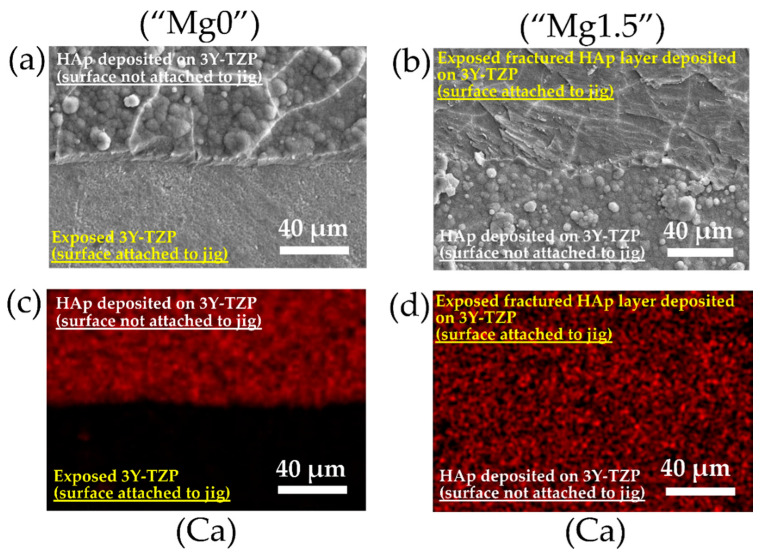
SEM images and EDX elemental mapping images of the surface of (**a**,**c**) “Mg0” and (**b**,**d**) “Mg1.5” solution treated HF-etched 3Y-TZP samples after the adhesive strength test.

In Figure 9, the SEM images and EDX elemental mapping images for Ca of fractured surfaces of “Mg0” and “Mg1.5” solutions treated HF-etched 3Y-TZP samples are shown, respectively. In the case of “Mg0” solution treated HF-etched 3Y-TZP samples, for the part of the surface attached to jig, HAp layer was removed and the surface of the 3Y-TZP was exposed after the fracture. While, in the case of “Mg1.5” solution treated HF-etched 3Y-TZP samples, for the part of the surface attached to jig, broken surface of HAp layer was observed after the fracture. Adhesive and cohesive failure are two common modes of failure between two different types of bonded materials. In the case of adhesive failure, the failure is at the interface while in the case of cohesive failure the material breaks itself which is the HAp layer in this study. Both the types of Ca-P solution-treated 3Y-TZP showed mixed modes of failures in different proportions. However, “Mg1.5” solution-treated HF-etched 3Y-TZP predominantly showed cohesive failure while “Mg0” solution-treated HF-etched 3Y-TZP showed adhesive failure.

Various studies have been reported the effect of Mg ion on the inhibition of Ca-P crystal growth [42,46,47,48]. Depending of the concentration of the Mg in the Ca-P particles, the Mg/Ca atomic ratio affects the particles morphology, crystallinity, and phase of the Ca-P particles. Incorporation of the Mg ion into the Ca-P particles results in the decrease of the Ca/P ratio. Mg ions replaces the Ca ions in the initial phase of amorphous Ca-P nuclei formation. This distorts the crystal structure by creating structural mismatch which prevent the growth of initial Ca-P nuclei into hydroxyapatite.

Barrere et al. reported the development Ca-P coating on Ti6Al4V samples having rough surface (Ra = 0.80 μm) from supersaturated SBF (SBF × 5) solutions which had five times the concentration of ions compared the conventional SBF in a CO_2_ atmosphere. Their study showed the effect of Mg ions on the precipitated Ca-P coatings. SBF with higher Mg concentration (SBF × 5, Mg × 8) resulted in the deposition of brushite or dicalcium phosphate dihydrate (DCPD). SBF solution without Mg (SBF × 5, Mg × 0) resulted in formation of carbonated apatite. At lower (SBF × 5, Mg × 3) and intermediate (SBF × 5) concentration of Mg resulted the deposition of amorphous carbonated Ca-P. It was concluded that Mg ions presence near the Ti6Al4V surface causes inhibition of the crystal growth of the apatite resulting in the formation of less crystallized tiny Ca-P globules. However, these tiny Ca-P structures causes stronger attachment of the Ca-P coating [49].

The present study was designed to quantitatively assess the effect of Mg ions on the enhancement of the adhesive strength of the HAp layer formed in the SBF. The physical parameters (temperature and pH) and concentration were controlled, and both types of Ca-P solution treatment either with or without Mg ions resulted in formation of slightly less crystallized HAp particles. Mg ions were incorporated in the Ca-P particles precipitated from “Mg1.5” Ca-P solution treatment, but Ma/Ca ratio was low to significantly affect the phase or crystallinity of precipitated Ca-P particles. However, Mg resulted in the decease of the particle size of precipitated particles and also led to the homogenous deposition of these particles on the 3Y-TZP substrate. Due the adequate deposition of the Ca-P particles, both of the solution treatments resulted in high HAp forming ability in the SBF. In the case of polished 3Y-TZP substrate due to the absence of any surface irregularity or micropores, both types of Ca-P solution treated samples resulted in the nucleation and growth of HAp crystals in the SBF on the surface without any strong adhesion. Whereas for the HF treated rough 3Y-TZP substrate, both types of Ca-P solution treatments resulted in the penetration of initially nucleated HAp crystals in SBF inside the surface irregularity or pores causing an enhancement of adhesive strength compared to polished samples. Furthermore, it is speculated that the sustained release of Mg ions from the particles deposited form “Mg1.5” solution near the vicinity of HF etched 3Y-TZP surface inhibited the growth of initially formed HAp crystal in the SBF. Being smaller in size these crystals were able penetrate more efficiently inside the surface pores or irregularities. These strongly penetrated crystals at the interface, fully developed into a dense HAp layer with increasing the immersion period in the SBF which increased the adhesion of the whole HAp layer. The results signify the importance of surface roughness and the contribution of Mg ions in the designed biomimetic Ca-P solution treatment on enhancing the adhesive strength of formed HAp layer in the SBF.

The authors acknowledge that there were limitations with the current study. Firstly, both types of Ca-P solution treatments showed similar HAp-forming ability in the SBF, despite having different morphology and composition of precipitated Ca-P particles. Further studies of the interface between the HAp and the 3Y-TZP substrate and cross-sectional analysis is to be done to clarify this point. However, in the context of in-vitro HAp-forming ability, the designed Ca-P solution treatments are found to be highly effective. Secondly, the authors applied one chemical treatment, i.e., HF etching. Both surface roughness and surface chemistry of substrate affect the HAp formation and its bonding ability to the substrate. The authors aim to further study the effect of different other surface treatments on the HAp formation. Lastly, the presented study clearly states the role of Mg ions on the bonding of HAp layer to the substrate formed in the acellular biomimetic environment as in SBF. However, a similar study has to be carried out in an ex vivo or tissue environment to further validate the point. These points will be clarified in further studies.

## 4. Conclusions

This work dealt with the fabrication of bioactive 3Y-TZP by biomimetic Ca-P solution treatment. HF etching was performed to enhance the surface roughness of the 3Y-TZP samples and then the samples were treated with two different types of Ca-P solutions, one solution contained Mg ions while other did not. Both types of solution treatments resulted in the deposition of Ca-P particles with different morphologies on the 3Y-TZP surface. Mg ions significantly reduced the size of Ca-P particles. Likewise, both these types of Ca-P solution treated 3Y-TZP samples showed HAp-forming ability in 1 day SBF immersion. The adhesive strength of the HAp layer to the 3Y-TZP surface was significantly low for the polished samples compared to HF-etched samples. Moreover, the adhesive strength also varied for the type of Ca-P solution treatments, Mg incorporated Ca-P solution treatment resulted in significantly higher adhesive strength of HAp layer on the HF-etched 3Y-TZP compared to Ca-P solution treatment with no Mg ions. It is considered that the release of Mg ions near the vicinity of the 3Y-TZP surface resulted in the formation of finer HAp crystal which strongly adhered to the pores on the roughened surface. A combination of HF etching and inclusion of Mg ions in the Ca-P solution treatment designed to induce HAp-forming ability resulted in sufficiently higher adhesive strength of the HAp layer to the surface of the substrate.

## Figures and Tables

**Table 1:** Name of solutions, concentration of the dissolved reagents in each Ca-P solution, and name of the precipitated calcium phosphate particles.

Name of Ca-P Solution	K_2_HPO_4_·3H_2_O [mM]	MgCl_2_·6H_2_O [mM]	CaCl_2_ [mM]	Name of Precipitated Calcium Phosphate Particles
“Mg0” solution	1.0	0	2.5	“Mg0” particles
“Mg1.5” solution	1.0	1.5	2.5	“Mg1.5” particles
